# Cross-sectional association between systemic metal concentrations and immune markers in patients with total joint arthroplasty

**DOI:** 10.3389/fimmu.2023.1130209

**Published:** 2023-03-13

**Authors:** Stephanie M. Peterson, Thomas J. O’Byrne, Peter C. Brennan, Paul J. Jannetto, Kevin D. Pavelko, David G. Lewallen, Maria Vassilaki, Hilal Maradit Kremers

**Affiliations:** ^1^ Department of Quantitative Health Sciences, Mayo Clinic, Rochester, MN, United States; ^2^ Department of Orthopedic Surgery, Mayo Clinic, Rochester, MN, United States; ^3^ Department of Laboratory Medicine & Pathology, Mayo Clinic, Rochester, MN, United States; ^4^ Department of Immunology, Mayo Clinic, Rochester, MN, United States

**Keywords:** cobalt, chromium, titanium, peripheral blood mononuclear cells, metal concentrations, total joint arthroplasty

## Abstract

Total joint arthroplasty (TJA) implants are composed of metal components. Although they are regarded safe, the long-term immunological effects of chronic exposure to the specific implant materials are unknown. We recruited 115 hip and/or knee TJA patients (mean age 68 years) who provided a blood draw for measurement of chromium, cobalt, titanium concentrations, inflammatory markers and systemic distribution of immune cells. We examined differences between the immune markers and the systemic concentrations of chromium, cobalt and titanium. CD66-b neutrophils, early natural killer cells (NK), and eosinophils were present in higher percentages in patients with chromium and cobalt concentrations greater than the median. The opposite pattern was observed with titanium where the percentages of CD66-b neutrophils, early NK, and eosinophils were higher in patients with undetectable titanium. Cobalt concentrations were positively correlated with a higher percentage of gamma delta T cells. Both chromium and cobalt concentrations were positively correlated with higher percentages of plasmablasts. Titanium concentrations were positively correlated with higher CD4 effector memory T cells, regulatory T cell count and Th1 CD4 helper cells. In this exploratory study, we observed altered distribution of immune cells in TJA patients with elevated systemic metal concentrations. Although these correlations were not strong, these exploratory findings warrant further investigation into the role of increased metals circulating in blood and its role in immune modulation.

## 1 Introduction

Total joint arthroplasty (TJA) of the hip and knee is a common procedure with over 7 million Americans currently living with artificial hip and knee implants (1). Implants are composed of metal components. Although they are regarded safe, the long-term immunological effects of chronic exposure to the specific implant materials are unknown.

Laboratory and histopathology studies indicate that metal debris at the bone-joint interface create an inflammatory reaction leading to osteolysis, loosening and implant failure (2–6). The immune response to metal debris depends upon the type, dose, and size of metal particles (6, 7). Despite the laboratory studies to date focused on local peri-implant tissues, the direction and the magnitude of the relationship between patients’ immune profiles and the systemic metal concentrations, or whether the systemic immune response depends upon the metal composition are unknown (8). We assessed the cross-sectional correlation of systemic metal concentrations and circulating immune cell populations.

## 2 Methods

We recruited 115 hip and/or knee TJA patients who had a clinical history of elevated serum metal concentrations. All patients provided consent to participate and had a blood draw (60 mL) for measurement of chromium, cobalt, titanium concentrations, inflammatory markers (C reactive protein [CRP], interleukin 6 [IL 6] and tumor necrosis factor alpha [TNF]) and systemic distribution of immune cells. Metal concentrations were assayed using inductively-coupled plasma mass spectrometry (ICP-MS) within an International Organization for Standardization Class-7 cleanroom in the Metals Laboratory (9, 10). Whole blood cobalt and chromium concentrations were measured using ICP-MS. Serum titanium was measured using Triple-Quadrupole ICP-MS. Inflammatory markers CRP, IL6 and TNF were measured using electrochemiluminescence and immunoturbidimetry.

Blood for peripheral blood mononuclear cells (PBMC) isolation was collected in a heparin or EDTA tube. PBMC provide a readily available and accessible resource for monitoring fluctuations in immune health due to systemic and environmental exposures. Here, PBMC were isolated and cryopreserved for downstream analysis. PBMC were analyzed with the Maxpar Direct Immune Profiling Assay (MDIPA; Standard BioTools, San Francisco, CA). This assay is composed of a tube with a lyophilized pellet of 30 antibodies labeled with heavy metal ions. These antibodies are detectable within the range of the ICP mass spectrometer contained within the Helios mass cytometer. After recovery from cryopreservation, viability was determined to be about 65 ± 20%, 3-4 million PBMC were added to the MDIPA assay tube and were further prepared for analysis using a previously established protocol (11). Data were acquired on a Helios mass cytometer/CyTOF and were analyzed with the Pathsetter analysis program (Fluidigm). The Pathsetter analysis program performs data cleanup and assessment of staining quality and viability. An analysis report is generated that identifies and classifies up to 37 unique cell populations from PBMC. These population counts include intact live cell percentages, and parent percentages for several populations including lymphocytes, monocytes, dendritic cells, and granulocytes. Optimally 300,000 intact live cells are analyzed by this approach and we found that mean viability of the PBMC was 75% after recovery from cryopreservation. In our dataset, 65 (56.5%) participants had 300,000 intact live cells, 11 (9.6%) had <200,000, and 39 (34%) had between 100,000 and 200,000 intact live cells. Two patients were excluded due to poor cell viability and an abnormally high plasmacytoid DC count. Thus, the final analyses included 113 patients.

**Figure 1:**
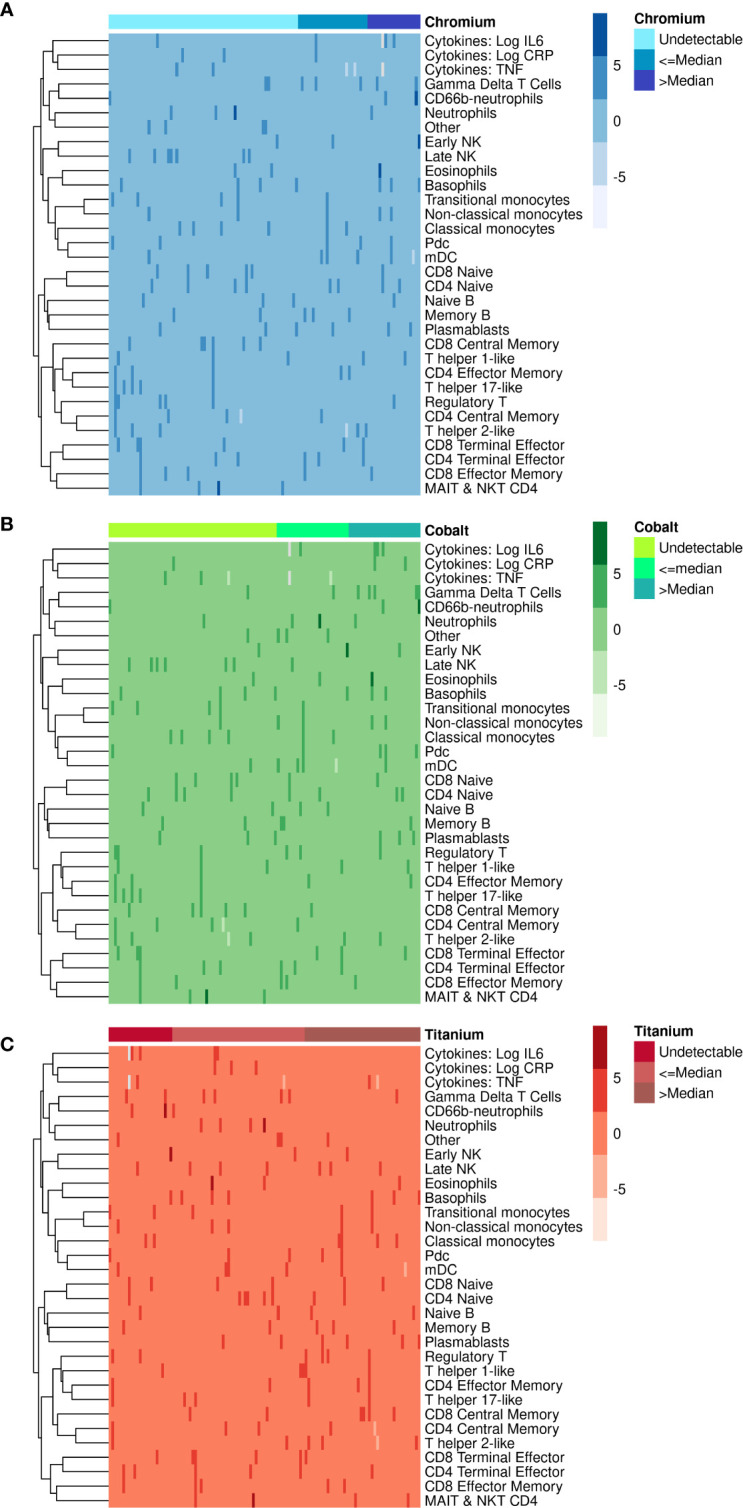
Heatmaps illustrating the association of immune cell populations with **(A)** chromium, **(B)** cobalt and **(C)** titanium concentrations in 113 TJA patients. Each column corresponds to an individual patient, and each row represents an immune cell population. Patients are categorized into 3 categories on top according to metal concentrations as undetectable, less than the median and more than the median. Color range in the heatmap is used to represent the abundance/scarcity of immune cells with dark cells indicating the most abundant and light cells the least abundant.

We created heat maps using the statistical package R to visualize differences between the immune markers and the systemic concentrations of chromium (**Figure 1A**), cobalt (**Figure 1B**) and titanium (**Figure 1C**). Different shades of color show the variability of values of each immune cell type (rows) for each of the 113 patients (columns). Patients were subcategorized into 3 groups by their metal concentrations as undetectable (<1 ng/mL), below or equal to the median, and above the median. Each cell represents the percent (i.e., count/live intact) of that immune cell population. Due to differing ranges of cell percentages, values were scaled to facilitate comparison ((X- mean X)/standard deviation of X).

We also examined the correlation of metal concentrations with cytokines (CRP, IL6, TNF) and immune cell subtypes (% of total cells) using Pearson correlation coefficients. Measurements of patients with “undetectable” metal concentrations (<1 ng/mL) were converted to numeric by using the highest numeric under the lower limit of quantification (e.g., 0.99 ng/mL). Due to the skewed distributions, we used the log transformation of all metal and cytokine concentrations. We did not apply multiple comparisons correction (12), as we investigated the associations to generate hypotheses for future studies. Statistical analyses were performed using SAS version 9.4 (SAS Institute; Cary, NC) and R version 3.6.2 (R Foundation for Statistical Computing; Vienna, Austria). Study protocols were approved by Mayo Clinic Institutional Review Board and participants provided written informed consent before participation.

## 3 Results

The mean age was 68 (standard deviation 10; range 43, 92) years, 56% were male and most participants except 2 were White (**Table 1**). A total of 81 (72%) patients had a history of hip TJA alone, 12 (11%) had a history of knee TJA alone, and 20 (18%) had both hip and knee TJA surgeries. The mean number of previous TJA surgeries was 2.6 (range 1, 8), including 63 (55%) patients reporting a history of revision surgeries.

**Table 1:** Charasteristics of the Study Population.

Characteristic	(N=113)
Mean (SD) age, years	68 (10)
Male sex, n (%)	63 (56%)
Race, n (%)	
Pacific Islander	2 (2%)
White	111 (98%)
Smoking history, n (%)	
Current smoker	4 (4%)
Previous smoker	35 (31%)
Never smoker	61 (54%)
Unknown	13 (11%)
TJA history, n (%)	
Hip	81 (72%)
Knee	12 (11%)
Both	20 (18%)
Years between first TJA surgery and study visit	
Mean (SD)	13.3 (5.7)
Median (Range)	12 (2.0, 31.0)
Metal concentrations (ng/mL)*	
Detectable chromium, n (%)	44 (39%)
Mean (SD)	3.7 (4.7)
Median (Range)	1.9 (1.1, 21.4)
Detectable cobalt, n (%)	52 (46%)
Mean (SD)	4.9 (4.3)
Median (Range)	3.6 (1.1, 24.6)
Detectable titanium, n (%)	90 (80%)
Mean (SD)	4.7 (5.1)
Median (Range)	3.0 (1.0, 35.0)

* The reference range for chromium and cobalt was <1 ng/mL and all of the patients with detectable chromium and cobalt concentrations had abnormal values. The reference range for titanium was <2 ng/mL and 72 of the 90 patients with detectable titanium concentrations had abnormal values.

Reference ranges for chromium, cobalt and titanium were <1 ng/mL, <1ng/mL and <2ng/mL, respectively. Forty-four (39%) patients had detectable chromium and 52 (46%) patients had detectable cobalt which were all above the reference range for normal (≥1 ng/mL). Ninety (80%) patients had detectable titanium concentrations and of these, 72 had abnormal values above the reference range for titanium (≥2 ng/mL). Among patients with detectable concentrations, median (range) concentration was 1.9 (1.1, 21.4) ng/mL for chromium, 3.6 (1.1, 24.6) ng/mL for cobalt, and 3.0 (1.0, 35.0) ng/mL for titanium.

The three heatmaps in **Figure 1** show that the percentage of most immune cell types were very similar (i.e., 0 on scaled percent), with only a few sporadic differences (darker shades of color). CD66-b neutrophils, early NK, and eosinophils were present in higher percentages when chromium (**Figure 1A**) and cobalt (**Figure 1B**) concentrations were greater than the median. The opposite pattern was observed with titanium where the percentages of CD66-b neutrophils, early NK, and eosinophils were higher in some patients with undetectable titanium (**Figure 1C**).

**Figure 2:**
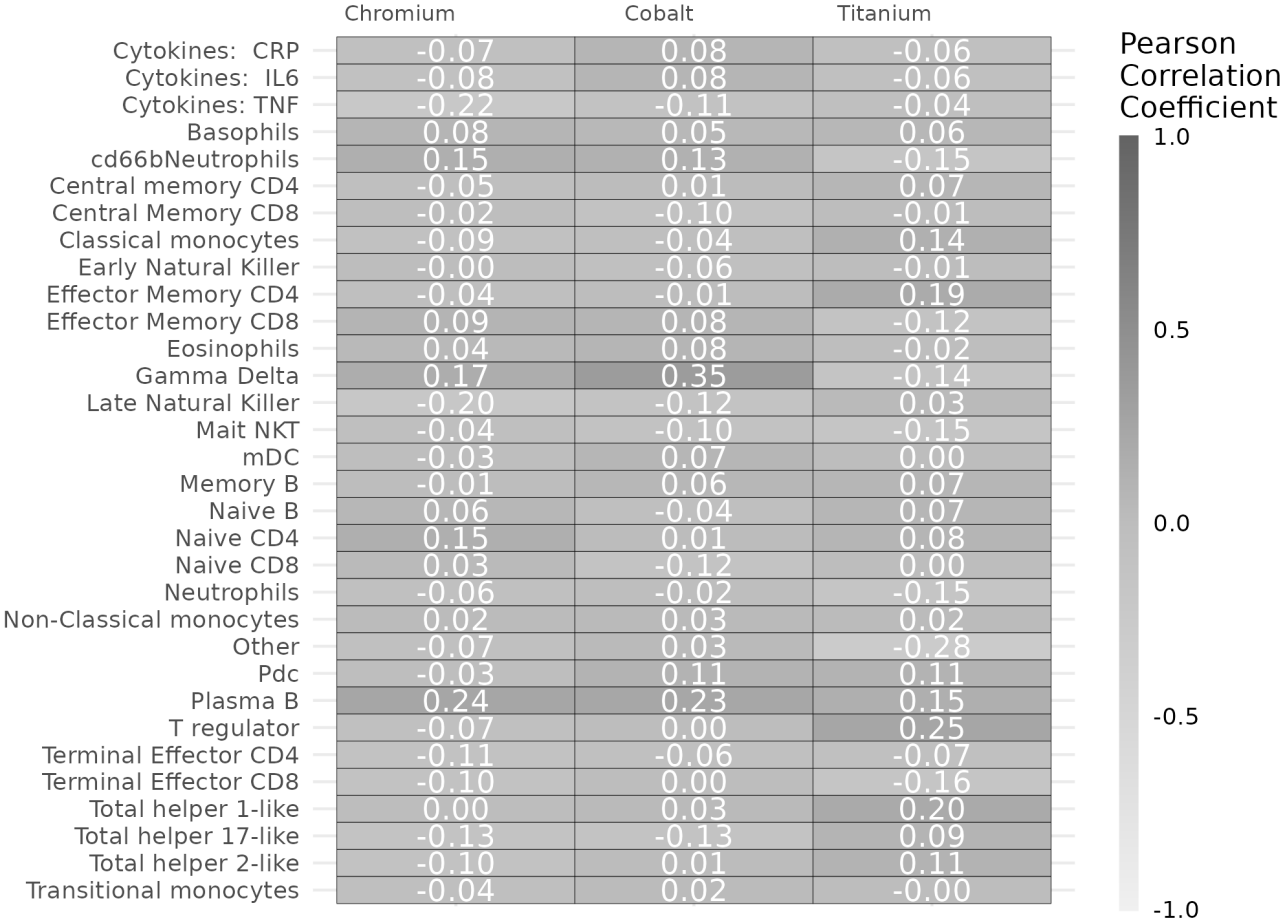
Pearson correlation coefficients of chromium, cobalt, and titanium concentrations with immune markers.

Cobalt concentrations were positively correlated with a higher percentage of gamma delta T cells (r = 0.35, *p* = 0.001) (**Figure 2**). Both chromium (r = 0.24, *p* = 0.01) and cobalt (r = 0.23, *p*= 0.01) concentrations were positively correlated with higher percentage of plasmablasts. Chromium concentrations were correlated with less late natural killer cells (r = -0.2, *p* = 0.04) and lower TNF concentrations (r = -0.22, *p* = 0.02). Titanium concentrations were positively correlated with higher CD4 effector memory T cells (r = 0.19, *p* = 0.04), regulatory T cell count (r = 0.25, *p* = 0.008) and Th1 CD4 helper cells (r = 0.20, *p* = 0.03).

## 4 Discussion

We observed altered distribution of immune cells in TJA patients with elevated systemic metal concentrations. The abundance of plasmablasts and gamma delta T cells with elevated cobalt-chromium concentrations suggests modulation of both innate and adaptive responses. Furthermore, abundance of effector memory CD4 cells and T-regulatory cells in patients with elevated titanium concentrations suggests that titanium may modify T-cell mediated immune responses. In the context of other lines of evidence, these exploratory findings warrant further investigation into the role of increased metals circulating in blood and its role in immune modulation.

Increased CD4 T helper cells and CD4 T-regulatory cell populations in patients with elevated titanium concentrations suggest that titanium may drive unique T-cell responses and influence downstream immune processes. Abundance of gamma-delta cells in patients with high cobalt concentrations suggests potential activation or mobilization of this innate cell type. Gamma-delta cells are typically tissue resident and cobalt exposure may trigger a response that promotes recruitment of these cells to sites of immune activation in the body. Higher percentages of plasmablasts with higher chromium and cobalt concentrations suggest increased reactivity of B-cells that are responding to specific stimuli or may represent recruitment of antibody secreting cells to novel sites in the body. Of interest is the potential for circulating metals to act as haptens that drive unique T helper cell responses as has been documented previously (13). Otherwise, studies in this area are mostly limited to immune responses around periprosthetic tissues (14). The combination of increased plasmablasts and CD4 T helper responses may point to the potential for hypersensitivity responses that are driven by circulating metals.

While the majority of subjects had undetectable levels of chromium and cobalt (61% and 54%, respectively), this is consistent with our unpublished internal reference range studies (~117 adult control subjects age range 23-89, 57 female, 60 male) where all (100%) subjects had concentrations <1 ng/mL which is the reference range for both chromium and cobalt in whole blood in patients without any implants. Otherwise, there is no reference range for TJA patients with metal implants who typically have higher concentrations (>1.0 ng/mL). That’s in part why we grouped patients by the median values instead of using the normal reference range cutoff (1.0 ng/mL) for patients without implants. The median value is where we typically see patients with implants who don’t have any signs of adverse local tissue reaction (ALTR)/adverse reaction to metal debris (ARMD).

Findings of this exploratory study should be interpreted considering potential limitations. Neither the metal concentrations nor the immune markers and cell distributions reflect the longitudinal trends or cause-effect relationship. We were not able to assess alternative sources for the metal concentrations, e.g., diet, mineral supplements, occupation. In addition, metallothioneins that modulate the availability of free metal ions were not available to our study. Future directions include performing a longitudinal study with serial measurements.

In conclusion, elevated systemic metal concentrations in TJA patients are associated with altered distribution of immune cells. Further research is warranted to elucidate the underlying mechanisms.

## Data availability statement

The de-identified data supporting the conclusions of this article will be made available by the authors, without undue reservation.

## Ethics statement

Study protocols were approved by Mayo Clinic Institutional Review Board and participants provided written informed consent before participation in the study.

## Author contributions

HMK provided the conception and design of the study. SP and TO’B performed the statistical analysis. SP, HMK, and MV drafted the manuscript. All authors assisted with the interpretation of data and critical revision of the manuscript for important intellectual content. HMK obtained funding. All authors contributed to manuscript revision, read, and approved the submitted version.
